# Supplementary material for: Twenty-seven ZAD-ZNF genes of Drosophila melanogaster are orthologous to the embryo polarity determining mosquito gene cucoid
Source: PLoS One. 2023 Jan 3;18(1):e0274716. doi: 10.1371/journal.pone.0274716 (PMC9810180; doi:10.1371/journal.pone.0274716)
Supplement: S2 Fig — The THAP domains from 9 THAP-containing proteins in D. melanogaster and a THAP-like fragment from CG17803 are shown in alignment here. The color code for each column is based on similarity of aligned amino acids, with black representing high similarity and white representing no similarity. The N-terminal THAP domain of CG6689 is absent in all other Cucoid orthologs including its most recent paralog, CG17803, which has incomplete THAP features. THAP is a zinc-coordinating DNA binding domain with a conserved C2CH structure and shares features with the DNA binding domain of the P element transposase [59,60]. THAP-domain-containing proteins have been found in human, D. melanogaster, and C. elegans [59]. In the nine D. melanogaster proteins that have this domain, only CG6689 and CG10431 belong to the ZAD-ZNF family. CG10431 is only distantly related to CG6689 and located on a different chromosome (2L), suggesting that even within the ZAD-ZNF family THAP domains evolved de novo. The THAP domain of CG6689 is encoded by the first two exons of this gene, which are only conserved in CG17803 (Fig 4). (PDF) [file pone.0274716.s006.pdf]

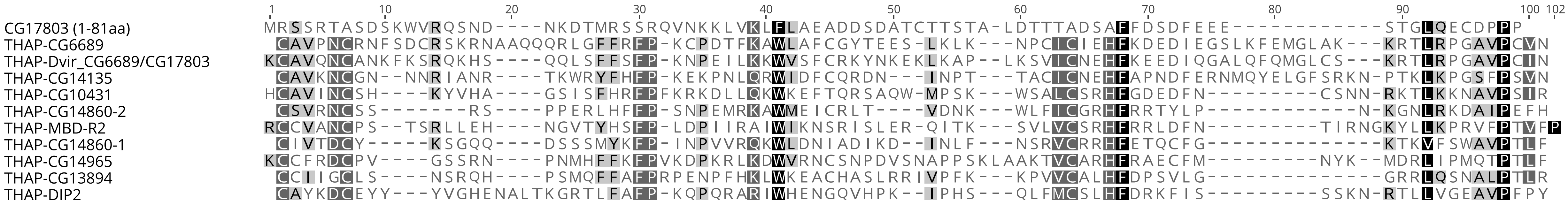
1 10 20 30 40 50 60 70 80 90 100 102
CG17803 (1-81aa) MRSSRTASDSKWVRQSND----NKDTMRSSRQVNKKLVKLFLAEADDSDATCTTSTA-LDTTTADSAFFDSDFEEE------------STGLQECDPPP
THAP-CG6689 CAVPNCRNFSDCRSKRNAAQQQRLGFFRFP-KCPDTFKAWLAFCGYTEES-LKLK---NPCICIEHFKDEDIEGSLKFEMGLAK---KRTLRPGAVPCVN
THAP-Dvir_CG6689/CG17803 KCAVQNCANKFKSRQKHS----QQLSFFSFP-KNPEILKKWVSFCRKYNKEKLKAP--LKSVICNEHFKEEDIQGALQFQMGLCS---KRTLRPGAVPCIN
THAP-CG14135 CAVKNCGN--NNRIANR----TKWRYFHFP-KEKPNLQRWIDFCQRDN---INPT---TACICNEHFAPNDFERNMQYELGFSRKN-PTKLKPGSFPSVN
THAP-CG10431 HCAVINCSH----KYVHA----GSISFHRFPFKRKDLLQKWKEFTQRSAQW-MPSK---WSALCSRHFGDEDFN---------CSNN-RKTLKKNAVPSIR
THAP-CG14860-2 CSVRNCSS-------RS----PPERLHFFP-SNPEMRKAWMEICRLT----VDNK---WLFICGRHFRRTYLP------------N-KGNLRKDAIPEFH
THAP-MBD-R2 RCCVANCPS--TSRLLEH----NGVTYHSFP-LDPIIRAIWIKNSRISLER-QITK---SVLVCSRHFRRLDFN---------TIRNGKYLLKPRVFPTVFP
THAP-CG14860-1 CIVTDCY-----KSGQQ----DSSSMYKFP-INPVVRQKWLDNIADIKD--INLF---NSRVCRRHFETQCFG--------------KTKVFSWAVPTLF
THAP-CG14965 KCCFRDCPV----GSSRN----PNMHFFKFPVKDPKRLKDWVRNCSNPDVSNAPPSKLAAKTVCARHFRAECFM---------NYK--MDRLIPMQTPTLF
THAP-CG13894 CCIIGCLS----NSRQH----PSMQFFAFPRPENPFHKLWKEACHASLRRIVPFK---KPVVCALHFDPSVLG--------------GRRLQSNALPTLR
THAP-DIP2 CAYKDCEYY---YVGHENALTKGRTLFAFP-KQPQRARIWHENGQVHPK--IPHS---QLFMCSLHFDRKFIS---------SSKN-RTLLVGEAVPFPY
