# Supplementary figures and images for: Twenty-seven ZAD-ZNF genes of Drosophila melanogaster are orthologous to the embryo polarity determining mosquito gene cucoid
Source: PLoS One. 2023 Jan 3;18(1):e0274716. doi: 10.1371/journal.pone.0274716 (PMC9810180; doi:10.1371/journal.pone.0274716)

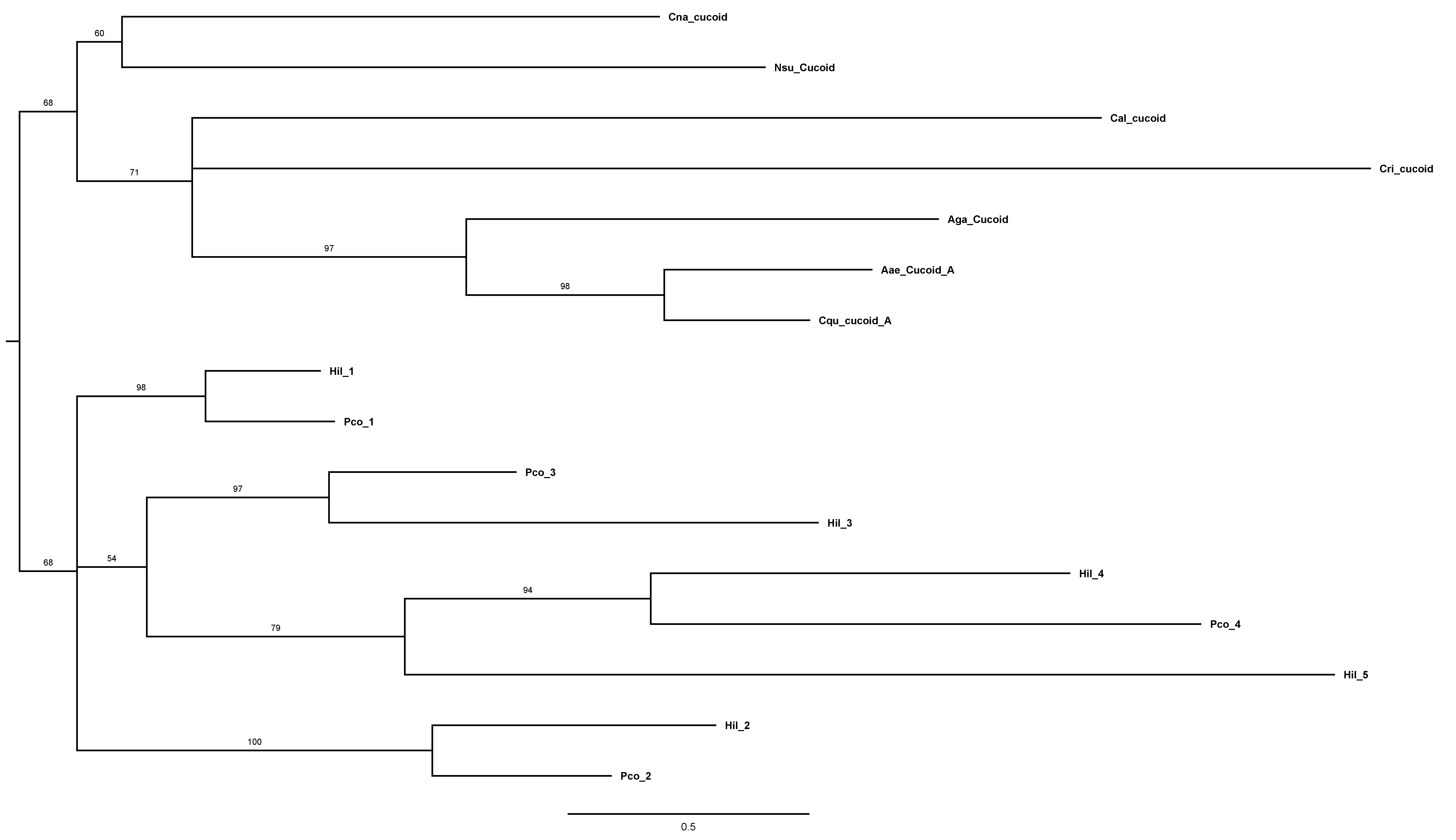

Supplement: S3 Fig — A phylogenetic tree with Cucoid orthologs in H. illucnes, P. coquilletti and lower flies was constructed based on an untrimmed alignment using 3 partitions inlucing ZAD, ZNF, and the other regions. Regions outside the ZAD and ZNF domains include diagnostic features useful for inferring orthology. This tree suggests that Hil_cucoid_1 to Hil_cucoid_4 are orthologous to Pco_cucoid_1 to Pco_cucoid_4, respectively. (TIF) [file pone.0274716.s007.tif]
